# Non-Parenteral Medications for Procedural Sedation in Children- A Narrative: Review Article

**Published:** 2015

**Authors:** Razieh FALLAH, Farzad FERDOSIAN, Ahmad SHAJARI

**Affiliations:** 1 Pediatric Neurologist, Department of Pediatrics, Growth Disorders of Children Research Center, Shahid Sadoughi University of Medical Sciences, Yazd, Iran; 2 Subspecialty in Pediatric Infectious Disease, Department of Pediatrics, Shahid Sadoughi University of Medical Sciences, Yazd, Iran; 3 Pediatric Nephrologist, Department of Pediatrics, Ali-ebn- Abitaleb School of Medicine, Islamic Azad University, Yazd Branch, Yazd, Iran

**Keywords:** Sedation, Children, Procedural sedation, Non-parenteral medications

## Abstract

Procedural sedation may be needed in many diagnostic and therapeutic procedures in children. To make pediatric procedural sedation as safe as possible, protocols should be developed by institutions. Response to sedation in children is highly variable, while some become deeply sedated after minimal doses, others may need much higher doses. Child developmental status, clinical circumstances and condition of patient should be considered and then pharmacologic and non-pharmacologic interventions for sedation be selected. Drug of choice and administration route depend on the condition of the child, type of procedure, and predicted pain degree. The drugs might be administered parenteral (intravenous or intramuscular) or non parenteral including oral, rectal, sublingual, aerosolized buccal and intranasal. The use of intravenous medication such propofol, ketamine, dexmedetomidine, or etomidate may be restricted in use by pediatric anesthesiologist or pediatric critical care specialists or pediatric emergency medicine specialists. In this review article we discuss on non-parenteral medications that can be used by non- anesthesiologist.

## Introduction

Many diagnostic and therapeutic procedures in children such as electroencephalography, computed tomography scan, magnetic resonance imaging, echocardiography, lumbar puncture, instrumentation (endoscopy, bronchoscopy,…), reduction of fracture, repair of laceration, abscess drainage, burn dressing

change, and chest tube or central line placement are needed. Procedural sedation and reduction of pain and anxiety outside the operating room have been emphasized (1).

Aims of procedural sedation include maintenance of patient’s safety and welfare, prevention of physical pain, discomfort and psychological trauma, anxiety and behavior control, amnesia maximization and reduce movement for safe performance of procedures. To make pediatric procedural sedation as safe as possible, protocols which specify a pre-sedation evaluation including a sedation plan, during the procedure and recovery monitoring, discharge and follow-up criteria, personnel credentialing, and an improvement of quality monitoring mechanism, should be developed by institutions. Providers of health care who carry out procedural sedation in children must have strong resuscitation and highly advanced pediatric life support skills including necessary training in assessing and management of the pediatric airway. Besides, they should have specific training in pediatric procedural sedation (2).

In this narrative review article, we have verified non-parenteral medications that can be used by nonanesthesiologist.

**Search strategy**

This narrative review study was conducted in 2014. Required data were collected through searching for key words included: “ Sedation” , “Children”,” Procedural sedation” , Non-parenteral medications “ and their Per¬sian equivalents using databases including Pub¬Med, OVID, Embase, Scopus, Magiran, scientiﬁc information database (SID), Google Search engine, and also Nelson Textbook of Pediatrics. To increase the confidence in the selection of articles, the reference lists of the articles, certain relevant journals and web site of up to date (www. uptodate.com ) in this field also were searched.

## Results

Levels of sedation are defined as follows:

**Minimal sedation: **Response of the patient is normal to verbal commands. Cognitive function and coordination might be impaired, but ventilatory and cardiovascular functions are not affected.

**Moderate sedation: **The patient has consciousness depression but can respond to verbal commands either alone or accompanied by light touch, maintains airway and enough ventilation without intervention and cardiovascular function is also maintained.

**Deep sedation: **The patient does not easily arouse but responds purposefully to noxious stimulation, may need help to maintain airway and adequate ventilation. Cardiovascular function is usually maintained (2, 3). The Joint Commission recommends that providers have the capability of one level deeper than the target depth for patients’ management (3). Response to sedation in children is highly variable, while some become deeply sedated after minimal doses, others may need much higher doses. In addition, differentiation of sedation levels may be hard or impossible during a given test or procedure for a given patient.

**Indications of procedural sedation in children**

There are no definite indications for the performance of children procedural sedation. It might be used for any procedure in which a child’s pain or anxiety is excessive and movement may prevent performance. The sedation need will vary highly with a child’s developmental or behavioral status. The targeted sedation depth and medications selection depends largely on the procedure performed the foreseen degree of pain, and patient factors (2, 3).

**Contraindications of procedural sedation in children**

There are no definite contraindications to children procedural sedation. Relative contraindications of procedural sedation include:

• Signs of a difficult airway or significant medical comorbidities [Patients with American Society of

Anesthesia (ASA) Classification III or higher and children with history of severe sleep apnea or airway

abnormality]

• True allergic reactions to sedative drugs

• Prior paradoxical reactions (patient becomes more agitated than sedated) with benzodiazepines or ketamine. 

• Children in whom the risk of sedation outweighs the Benefit Risk of aspiration might be increased in children with severe gastroesophageal reflux disease and acute or chronic decreased gastrointestinal motility and continuation of precautions for aspiration is necessary at all times.

**Pre-sedation evaluation**

**A: Taking a history**

Medical history about medical illnesses ( respiratory, cardiovascular, and neurologic systems), snoring or central or obstructive sleep apnea, previous exposure to sedation or general anesthesia, drug and food allergies, current medications, family history of an adverse reaction to sedation, analgesia, or general anesthesia, and the time and content of their last oral intake should be taken. Review of systems that evaluate cardiac, pulmonary, renal, bowel and hepatic function should also be done (4).

**B: ASA Classification**

Before sedation, sedation risk assessment, airway assessment and ASA classification should be given to each patient as follows: 

Class I: A normally healthy patient 

Class II: A patient with mild to moderate systemic disease with no functional limitations (mild chronic renal failure, iron deficiency anemia, mild asthma, controlled diabetes mellitus)

Class III: A patient with severe systemic disease with functional limitations but is not incapacitating (moderateto- severe asthma, poorly controlled diabetes mellitus, pneumonia, congenital heart disease, cystic fibrosis) 

Class IV: A patient with incapacitating systemic disease that is a constant threat to life (severe bronchopulmonary dysplasia, advanced cardiac disease) Class V: A moribund patient who is not expected to survive 24 hours, with or without surgery (septic shock, severe trauma) 

Class VI: A clinically dead patient being maintained for harvesting organs. Children with ASA classes I and II are generally reasonable candidates for mild, moderate, and deep sedation by the personnel other than anesthesiologists, outside of the operating room. Those patients with ASA classes III, IV, and V, special needs, or airway abnormalities warrant consultation with a pediatric anesthesiologist or pediatric critical care specialists or pediatric emergency medicine specialists (2).

**C: Obtaining written informed consent from the primary caregiver**

Before performing procedural sedation, the clinician should be ensured that monitoring devices are present and functioning properly and that oxygen, oxygen delivery devices, suction catheters and apparatus, pediatric airway equipment (laryngoscope with appropriately sized blades, endotracheal tubes and laryngeal mask airway), an emergency cart with appropriate medications, and a defibrillator are immediately available and obtain informed consent from the patient or the primary caregiver and the specific medications that will be administered. Potential routes of administration for procedural sedation and alternatives such as regional anesthesia or general anesthesia, potential side effects (vomiting, respiratory depression, laryngospasm, emergence reaction), and the likely duration of sedation should be explained. Because depression of consciousness is a continuum and responses to medications vary, clinicians must be able to deal with complications in patients whose level of sedation becomes deeper than intended or who experience an adverse reaction to medication (5, 6).

**D: Fasting**

ASA suggests that children undergoing sedation for scheduled elective procedures outside of the operating room, to fast as follows: two hours for clear liquids, four hours after breast feeding, six hours for solid foods, formula, or non- human milk. However, there is little evidence that this approach actually prevents aspiration(5).Based on the National Institute for Health and Clinical Excellence guidance, fasting is not needed for sedation with 50% nitrous oxide (in oxygen) alone and for moderate sedation where the child maintains verbal contact. Fasting based on the ASA guideline is advised for patients with moderate sedation where the child might not maintain verbal contact and deep sedation (6).

**E: Performing procedural sedation**

Performing of sedation requires at least two individuals, typically an advanced practice clinician (eg, physician, physician’s assistant, advanced practice registered nurse, nurse anesthetist) and an assistant. At least one present person should be trained in advanced pediatric life support and be skilled in airway management and cardiopulmonary resuscitation.

**1: Monitoring**

Vital signs (pulse rate, respiratory rate and blood pressure) should be recorded before starting the procedure, after description of sedative drugs, at completion of the procedure, during early recovery and at completion of recovery (3). Level of consciousness, drugs that is administrated, and any complications that may be occurred, should be monitored continuously. Monitoring equipment appropriate to the degree of sedation should be ready for use as follows:• Mild sedation: Pulse oximetry and heart rate• Moderate or deep sedation: Pulse oximetry, capnography or continuous visual monitoring of breathing (face, mouth, and chest wall movement), heart rate, respirations, and blood pressure 

**2: Vascular access**

Children that receive deep sedation should have an intravenous catheter in place for administration of

multiple doses of drugs or for resuscitation, if needed.

Although desirable intravenous access is mandatory neither for lighter levels of sedation nor when sedative agents are given by oral, nasal, rectal, or intramuscular routes. If the procedure is performed without an intravenous catheter, equipment and personnel capable of establishing vascular access should be immediately available

**3:** **Selection of procedural sedation intervention in children**

Developmental status of child, clinical circumstances and condition of patient should be considered and then pharmacologic and nonpharmacologic interventions for sedation are selected (1).

**Nonpharmacologic interventions**

For many children, non-pharmacologic interventions use (behavioral and cognitive approaches) might prevent the need for procedural sedation. In conscious situations of the patient during the sedation, behavioral and cognitive approaches are complementary to pharmacologic interventions and must be used. These techniques aid to reduce preprocedural agitation which lets an easier transition to sedation as well, may decrease the amount of medication needed for effective sedation, and may cut adverse events frequency, including emergence phenomena. Behavioral treatments include desensitization techniques, distraction (non-nutritive sucking in infants, bubble blowing, party blowers, listening to a book, counting, interactive toys, playing music through headphones, video games, and videotapes), reinforcing coping skills, positive reinforcement, and relaxation techniques (2, 7). Parental involvement increases the effect of behavioral pain management if the parent is properly prepared for a positive role and managing of parental anxiety is an important element in allaying a child’s fears (7).

**Pharmacologic medications for pediatric procedural sedation**

Drug of choice and administration route depend on the condition of the child, type of procedure, and predicted pain degree. Non- painful procedures that require immobility of the child can be done with sedation alone.

However, painful procedures require analgesia as well as sedation. Drugs might be administered parenteral (intravenous or intramuscular) or non- parenteral including oral, rectal, sublingual, aerosolized buccal and intranasal.

The use of intravenous medication such propofol, ketamine, dexmedetomidine, or etomidate may be

restricted to use by anesthesiologists or other specialists.

We discuss non-parenteral medications here.

**Non-parenteral sedative drugs**

* Promethazine

Promethazine is a cheap and easily available antihistamine with antiemetic properties, which might

be used for sedation induction, especially in regions that chloral hydrate is unavailable (8, 9).

In a study in Yazd, Iran, adequate sedation and successful electroencephalography recording were obtained in 70% of children with ASA class I or II who received 1 mg/kg of promethazine (8).

* **Chloral hydrate**

Chloral hydrate is a non-opiate, non-benzodiazepines sedative hypnotic drug, used for pediatric sedation in dosage of 40-100 mg/kg for years especially for diagnostic imaging in infants and in younger than threeyear-old children image. It can be administered orally or rectally and action onset of a single dose of chloral hydrate takes up to 30 minutes and up to 60 minutes while re-dosing is necessary. The onset and degree of sedation may be undependable with the rectal route. Time to discharge is 5 to 10 days with the longer time occurring in children who need re-dosing (8-13). The drug is contraindicated in children with kidney, liver, or cardiovascular disease. It should not be administered by oral in children with gastritis, esophagitis, or peptic ulcers in view of mucosal irritation. Side effects include gastrointestinal irritation and vomiting with oral use, excessive sleepiness and prolonged sedation, hallucinations and paradoxical agitation, airway obstruction, respiratory depression and oxygen desaturation, postprocedural bradycardia, especially in young term and preterm infants and tachyarrhythmia with excessive and higher dosage (14, 15). Monitoring procedural sedation protocols must be followed for all patients receiving chloral hydrate. As there is no consistent dose, below which complications do not happen (3) and in young infants, up to 12 hours observation after chloral hydrate sedation may be necessary prior to safe discharge (16). In two other studies in Yazd, Iran, combination of chloral hydrate and antihistamines (hydroxyzine or promethazine) was an effective and safe sedation regimen in children (9, 10). Some countries have put aside chloral hydrate from their national health formularies due to potential carcinogenicity even if the risk of cancer from a single dose is inconclusive (17).

* **Midazolam**

Midazolam is a short-acting water soluble benzodiazepine with anxiolytic and amnestic properties that can be used orally, rectal, sublingual, aerosolized buccal, intranasal, intravenous or intramuscular that dosage, effect duration and onset of action depends on child age and administration method and action duration of midazolam is shorter than pentobarbital or chloral hydrate (3, 11, 13, 17-19). Intranasal rout has the most rapid onset of action and shortest recovery time in comparison to oral, rectal or sublingual administration. Rectal midazolam is less dependable and may arouse patient discomfort. Since, intranasal midazolam might be irritant in a few patients, administration of 10 mg per puff of lidocaine spray, one minute before prescription of intranasal midazolam can reduce nasal mucosal irritation (20). Ampoule of midazolam can be used orally or intranasal when oral or intranasal midazolam is not available (18). Injectable midazolam given orally in dose of 0.75 mg/kg as a premedication was effective in Sheta et al. study (21). Dosage of oral midazolam in children is 0.5 – 1 mg/kg (22) and dose of intranasal midazolam is 0.2-0.5 mg/kg (11, 23). Action onset of oral, sublingual and intranasal midazolam is similar (5-10 minutes), but sedation duration of intranasal midazolam is shorter (20-30 minutes) and recovery time of oral or sublingual is approximatel 60 minutes. Unlike barbiturates or chloral hydrate, midazolam is not associated with prolonged symptoms of ataxia, sleepiness, or irritability (24, 25). Common side effects include respiratory depression or apnea, especially when used with opioids or other sedative drugs, and paradoxical reactions (hyperactivity, aggressive behavior and inconsolable crying). Midazolam has mild negative inotropic effects. It must be administered with caution in children with underlying myocardial depression. Respiratory depression or apnea and paradoxical reactions can be reversed with flumazenil. Nevertheless, flumazenil must not be used in patients with seizure disorders or in those who receive benzodiazepines on a chronic basis due to the risk of precipitating seizures or withdrawal symptoms, respectively (3).

* Melatonin

Melatonin is an indoleamine and useful oral naturalsleep agent. Its main role is modulation of the circadian rhythm of, sleep, and can be used as a safe and effective sedative drug in procedures of children. In two studies in children, adequate sedation and successful electroencephalography recording was achieved in 73% of 1-8 year old ones who did not naturally sleep and immobilized and were in ASA class I or II (18, 26, 27). 

* Short-acting barbiturates

Pentobarbital, methohexital, and thiopental are shortacting barbiturates that induce sedation by inhibiting gamma-aminobutyric acid receptors in the central nervous system and pentobarbital has the best efficacy and fewest adverse effects. Dosage of orally or rectally pentobarbital is 3 to 6 mg/kg in less than four year old children and 1.5 to 3 mg/kg in four year old and older ones and maximum single dose is 100 mg. Methohexital and thiopental can be used rectally. Dosage of rectal methohexital is 25 mg/kg with maximum 500 mg. It can cause seizures in children with epilepsy and should be avoided in these patients. Dosage of rectal thiopental is 5 to 30 mg/kg with maximum 700 mg. Common side effects of short-acting barbiturates are respiratory depression, hypotension, and myocardial depression. Pentobarbital has the lowest risk of respiratory depression and rectal thiopental has the highest risk. The potential for airway compromise rises while barbiturates are used combined with other sedatives or opioids. Prolonged sleepiness is common after pentobarbital administration. Absolute contraindications of use of barbiturates are porphyria and its relative contraindications include cardiac, liver, or renal insufficiency and the drugs should be avoided in patients with hemodynamic instability or heart failure. Methohexital may cause seizures in children with epilepsy. It must not be used in such patients (28, 29).

* Dexmedetomidine

Dexmedetomidine is a selective alpha-2 adrenergic receptor agonist that can be used by intranasal, oral or buccal routes for pediatric sedation and it causes minimal respiratory depression. In healthy children, it can be used as a safe and effective and is the preferred drug for sedation induction in diagnostic imaging (30, 31). Effectiveness of 0.5 mg/kg oral midazolam or 1.5 mcg/ kg of intranasal dexmedetomidine in sedation induction was equal, but, intranasal dexmedetomidine was accompanied with less post-procedural pain scores (32) and perioperative administration of 3 to 4 mcg/kg buccal dexmedetomidine showed moderate sedation without respiratory complications (33, 34) .

Side effects of dexmedetomidine include:

• Hypertension might be seen in up to 5% of patients that take continuous infusion of dexmedetomidine and typically resolves without specific treatment.

• Moderate bradycardia and hypotension might occur in up to 30% of children who receive

dexmedetomidine continuous infusion and hypotension is usually reversed with a fluid bolus. Dexmedetomidine is contraindicated in children who receive medicines with rate slowing action on the AV node (digoxin, nifedipine ), cardiac conduction system pathology, and those in whom increased pulmonaryartery pressure or decreased cardiac output are unlikely to be well tolerated (eg, right-sided heart failure, septic shock) and relative contraindications include children who are debilitated, inadequately hydrated, or have reduced cardiac output (35, 36).

* Ketamine

Ketamine is a derivative of phencyclidine with the impact that is either present or absent and provides sedation, analgesia, amnesia, and immobilization, while usually preserve upper airway muscle tone, airway protective reflexes, and spontaneous breathing and the drug can be administered intranasal or oral for children premedication (37-39). 

**F: Discharge criteria**

Returning to the level of responsiveness in handicapped young infants or children, should be observed before sedation. Monitoring must continue until the child meets the criteria for safe discharge including airway patency and stable cardiovascular function, easy arousability with intact protective reflexes, talk ability, unaided ability to sit up and maintain wakefulness, enough hydration with any nausea or vomiting management and also right management of any continued pain (40). The minimum observation duration of infants, include:

• All term or pre-term infants with post conceptual ages (PCA) ≤45 weeks : 12 hours

• Pre-term infants with PCA 46 to 60 weeks and important comorbidities : 12 hours

• Healthy pre-term infants with PCA 46 to 60 weeks: 6 hours (12 hours if prescribed opioids or other medications with notable respiratory depressant effects) (41, 42).
